# SARS-CoV-2 Delta and Omicron Variants Surge in Curitiba, Southern Brazil, and Its Impact on Overall COVID-19 Lethality

**DOI:** 10.3390/v14040809

**Published:** 2022-04-14

**Authors:** Douglas Adamoski, Valter Antonio de Baura, Ana Carolina Rodrigues, Carla Adriane Royer, Mateus Nóbrega Aoki, Marcel Kruchelski Tschá, Ana Claudia Bonatto, Roseli Wassem, Meri Bordignon Nogueira, Sonia Mara Raboni, Bernardo Montesanti Machado de Almeida, Edvaldo da Silva Trindade, Daniela Fiori Gradia, Emanuel Maltempi Souza, Jaqueline Carvalho de Oliveira

**Affiliations:** 1 Brazilian Biosciences National Laboratory (LNBio), Brazilian Center for Research in Energy and Materials (CNPEM), Campinas 13083-970, Brazil; douglas.adamoski@gmail.com; 2 Department of Biochemistry and Molecular Biology, Federal University of Paraná, Curitiba 81530-000, Brazil; valter.baura@gmail.com; 3 Department of Genetics, Universidade Federal do Paraná, Curitiba 81531-980, Brazil; ana.acr.rodrigues@gmail.com (A.C.R.); carladriane@gmail.com (C.A.R.); anacbonatto@gmail.com (A.C.B.); wassem@ufpr.br (R.W.); danielagradia@ufpr.br (D.F.G.); 4 Carlos Chagas Institute, FioCruz, Curitiba 81310-020, Brazil; mateus.aoki@fiocruz.br; 5 Molecular Biology Institute of Paraná, Curitiba 81310-020, Brazil; marcelkt@ibmp.org.br; 6 Virology Laboratory, Complexo Hospital de Clínicas, Universidade Federal do Paraná, Curitiba 80060-900, Brazil; meribor@ufpr.br (M.B.N.); sraboni@ufpr.br (S.M.R.); 7 HiLab Laboratories, Curitiba 81270-185, Brazil; bernardo@hilab.com.br; 8 Department of Cellular Biology, Federal University of Paraná, Curitiba 81530-000, Brazil; edstrindad@gmail.com

**Keywords:** coronavirus, environment, public health, diagnosis, diagnostic techniques and procedures, genotyping, SARS-CoV-2, omicron, vaccination

## Abstract

Screening efforts and genomic surveillance are essential tools to evaluate the course of the COVID-19 pandemic and assist the public healthcare system in dealing with an increasing number of infections. For the analysis of COVID-19 cases scenarios in Curitiba, Paraná, Brazil, we performed a diagnosis of positive cases, coupled with genotyping, for symptomatic and asymptomatic members of the Federal University of Paraná. We achieved over 1000 samples using RT-qPCR for diagnosis. The posterior genotyping allowed us to observe differences in the spread of strains in Curitiba, Brazil. The Delta variant was not associated with an infection wave, whereas the rapid Omicron variant spread became dominant in less than one month. We also evaluated the general vaccination coverage in the state, observing a striking reduction in lethality correlated to the vaccinated fraction of the population; although lower lethality rates were not much affected by the Omicron variant wave, the same effect was not translated in the number of infections. In summary, our results provide a general overview of the pandemic’s course in Paraná State and how there was reduction in lethality after a combination of multiple infection waves and a large-scale vaccination program.

## 1. Introduction

Despite the considerable efforts in vaccination to control the COVID-19 pandemic worldwide, the emergence of highly infectious variants, such as Alpha, Beta, Gamma, Delta, and recently, Omicron variants of concern (VOCs) [1] is a matter of public health concern. In Brazil, the Gamma variant (B.1.1.28/P.1 lineage) emerged in Manaus in late January 2021, surpassing Zeta (B.1.1.28/P.2 lineage), a predominant variant of interest (VOI) at that time, in less than three months [2].

The Delta variant (B.1.617.2 lineage) was initially detected in India in October 2020 [1], and responsible for severe burden on its healthcare system, especially in regard to medical equipment and supplies, entailing in a shortage of hospital beds and oxygen supply for critically ill patients [3]. This variant spread worldwide, even to countries with high vaccination rates [4]. In Brazil, the first confirmed case occurred on 26 April 2021 [5].

The Omicron variant (B.1.1.529 lineage) is currently the main object of worldwide concern. It was identified for the first time on 14 November 2021, in South Africa; due to numerous mutations, this variant has shown to have increased transmissibility and the potential to partially suppress infection or vaccine-induced immunities [6]. The particular constellation of Omicron mutations led to an increased doubling time compared with previous variants, especially considering the combination with immune evasion [7].

To perform genomic surveillance, we analyzed 1299 positive samples from 29 June 2020 to 31 January 2022, collected in the COVID-19 detection service of LIGH (Laboratory of Immunogenetics and Histocompatibility), at Federal University of Parana (UFPR) in Curitiba, Brazil. 1118 samples were successfully genotyped. Vaccination profiles for Paraná state were obtained from Brazilian DATASUS, on 8 February 2022. The UFPR’s Complexo Hospital de Clínicas Research Ethics Committee approved the study (CAAE: 31687620.2.0000.0096), and all participants signed an informed consent form. All data used in the plots are available in Supplementary Table S1.

## 2. Materials and Methods

Samples processed came mainly from two distinct sources, impacting the material received: (1) nasopharyngeal swab in viral transport medium from CHC-UFPR, mainly from healthcare workers, as previous described [8] or (2) crude saliva samples collected in LIGH-UFPR continuous symptomatic testing or single-day asymptomatic testing, as previous described [2]. For nasopharyngeal swab samples, 100 µL of carrier VTM were used to perform the extraction. For saliva, samples were processed as previously described [2]. Briefly, after initial homogeneization, samples settled for 30 min or centrifuged for 2 min (2000× *g*). As in nasopharyngeal swabs, 200 µL from each saliva sample were pooled in groups of 5 when the donor was asymptomatic or processed individually when the donor was reported symptomatic. We performed RNA extraction by using an automated magnetic EXTRACTA–RNA and DNA Viral kit (Loccus Biotecnologia, Sao Paulo, Brazil). We performed amplification in 3 ways: on a QuantStudio5 instrument (ThermoFisher Scientific, https://www.thermofisher.com, accessed on 16 March 2022) using AllPlex nCov-2019 reverse transcription PCR Master Mix Kit (SeeGene, Seoul, South Korea, performing 45 cycles), Molecular SARS-CoV-2 EDx (Bio-Manguinhos/FioCruz, Rio de Janeiro, Brazil, performing 40 cycles), or KIT BIOMOL OneStep/COVID-19 (IBMP, Curitiba, Brazil, performing 40 cycles). All molecular kits were used accordingly to the manufacturer instructions. Multiple kits were used due to the issues with supply chain and the imperative need to keep routine testing.

As performed in a previous study [2], positive samples were evaluated using probe-based genotyping systems to detect VOCs. At first, Vogels et al.’s [8] multiplex approach was applied to detect Spike Δ69–70 and ORF1a Δ3675–3677 deletions as an outcome for distinguishing Alpha, Beta, Gamma, Delta, Omicron, or other wild-type variants [9]. Classified samples were re-analyzed with two allelic discrimination TaqMan assays (Thermo Fisher Scientific Inc., Waltham, MA, USA): N501Y (ANPRYZA), and P681R (CVEPRY4). All assays were performed using GoTaq^®^ Probe 1-Step RT-qPCR System (Promega, Madison, WI, USA) on a QuantStudio5™ instrument (Thermo Fisher Scientific Inc., Waltham, MA, USA). The N501/681R+ profile is consistent with the Delta variant, whereas the Y501/non-detection 681 profile is consistent with the Omicron variant [10]. We confirmed the presence of Delta (plus T95I and G142D, AY.1/AY.4 strains) and Omicron variants through partial genome-sequencing of the S gene from 23 samples, selected to validate the probes assay, using the BigDye Terminator v.3.1 Cycle Sequencing Ready Reaction kit in an AB1 3500xL automated sequencer (Thermo Fisher Scientific Inc., Waltham, MA, USA).

Epidemiological data from Paraná state were obtained from Secretaria Estadual da Saude (https://www.saude.pr.gov.br/Pagina/Coronavirus-COVID-19), accessed on 7 February 2022. Epidemiological data from Brazil were obtained from Ministerio da Saúde (https://COVID.saude.gov.br/), accessed on 31 March 2022. Vaccination data were obtained from OpenDATASUS (https://opendatasus.saude.gov.br/dataset/COVID-19-vacinacao), accessed on 31 March 2022.

## 3. Results and Discussion

**Figure 1:**
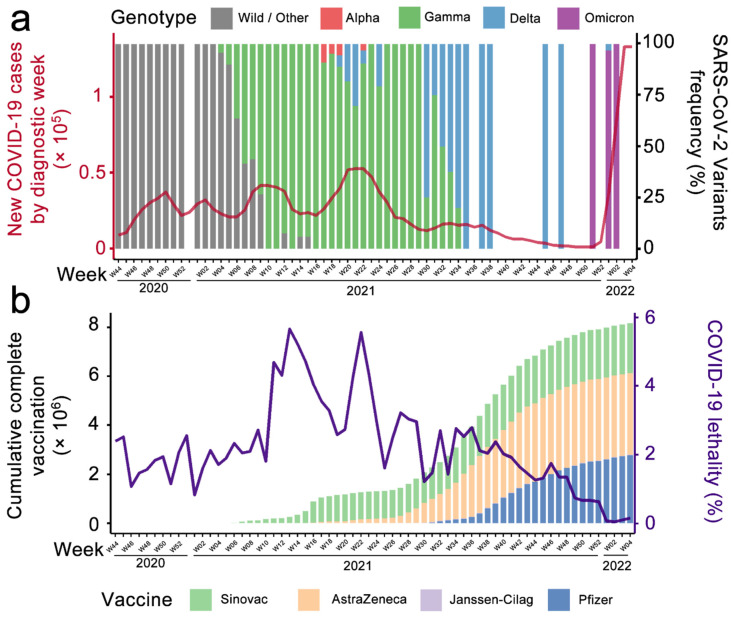
SARS-CoV-2 variants, number of cases, lethality, and vaccination in Paraná State, Southern Brazil. (**a**) New COVID-19 cases (red line, left axis) are grouped by epidemiological week. Colored bars (right axis) indicate the frequency of Wild, Alpha, Gamma, Delta, and Omicron variants in Curitiba, Paraná. (**b**) The cumulative complete vaccination (left axis) in Paraná State, Brazil, in millions of habitants, is stratified by vaccine manufacturer: Sinovac (inactivated virus, two doses), AstraZeneca (Adenovirus, two doses), Janssen-Cilag (Adenovirus, two doses, despite manufacturer single-dose regimen suggestion), and Pfizer (mRNA, two doses). COVID-19 lethality (right axis) is defined as deaths or registered cases in each epidemiological week.

The first detection of the Delta variant among our collected samples occurred on 13 May 2021 (week 19, 2021), with 17.4% incidence during this month. The reduction in the Gamma variant in the following months resulted in the total predominance of Delta by September (Week 35, 2021, Figure 1a). Although this replacement was expected, as it was observed in other countries in Asia, North America, and Europe [11], the surge of the Delta variant in Brazil was not accompanied by infection and lethality waves [12]. In Paraná State, the predominance of Delta cases did not lead to an increase in infections per week unlike Gamma, which was the most prevalent variant in previous peaks, seen in weeks 10 and 21 (Figure 1a). This could be partially explained either by the advance of vaccination, with more than 18% of Paraná’s total population completely vaccinated at that point, or by the close wave of Gamma infections, which was still ongoing when the first Delta cases were confirmed in the state (Figure 1b). 

Following other countries, the surge of Omicron in Brazil happened incredibly fast, quickly displacing Delta. This variant represented a significant increase in the number of infections, but maintained a proportionally low increase in the number of deaths [13]. Cases attributed to the Omicron variant started to circulate in week 51 (2021), with the unprecedented mark of 142,737 cases (1.3% of the state’s total population) in a single week. However, COVID-19’s lethality had already decreased since, as of early 2022, more than 70% of the population had the complete vaccination for SARS-CoV-2. The low intrinsic pathogenicity of the Omicron variant [13] and the high seroprevalence of the population (caused by either previous infections and/or vaccination) led to a two times higher incidence of cases in the state (Figure 1a), but with the lowest lethality rate of the entire pandemic (Figure 1b).

**Figure 2:**
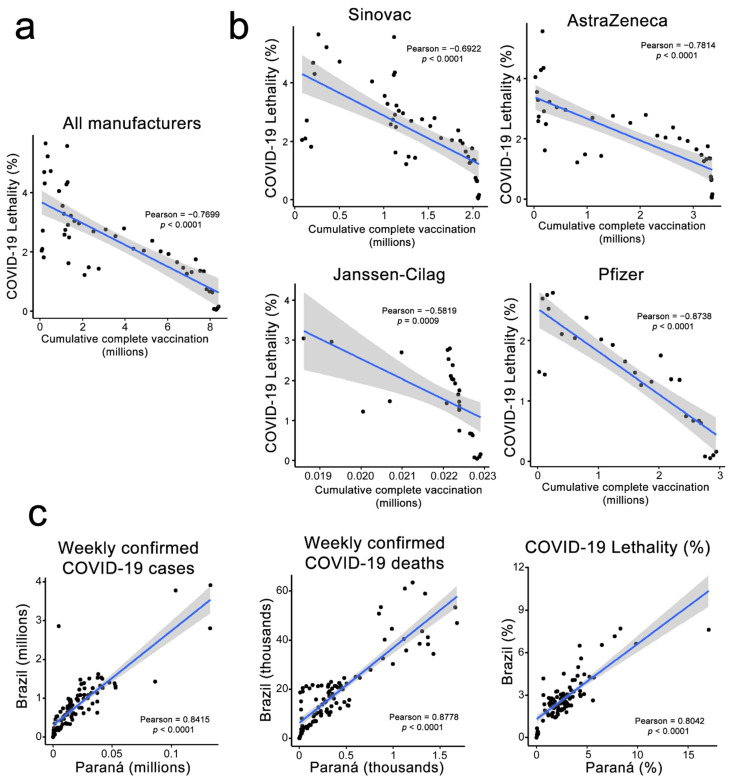
Correlation between vaccination rates and SARS-CoV-2 lethality in Paraná State and comparison with general country infection rates. (**a**) Correlation between cumulative two-doses complete vaccination cycle reported weekly using any vaccine in Paraná state (*x*-axis) and COVID-19 disease lethality (*y*-axis). (**b**) Same as (**a**), but stratified by each vaccine manufacturer. (**c**) Correlation between Paraná state epidemiological scenario (*x*-axis) and Brazil one (*y*-axis) for weekly confirmed COVID-19 cases (left), COVID-19 confirmed deaths (middle), and COVID-19 lethality (right). Blue line represents a linear regression; values derive from Pearson correlation and its *p*-value.

To further confirm the evidence of the correlation between the complete vaccination cycle of the population and the reduction in disease lethality, we correlated cumulative vaccination and disease lethality, measured on a weekly basis (Figure 2a). A negative correlation (Pearson = −0.7699) with a significative *p*-value (<0.0001) denotes the reduction in disease impact in a vaccinated population. The same scenario is observed when we stratify the data by each vaccine manufacturer (Figure 2b), with the worst signal (Pearson = −0.5819) for Janssen-Cilag, due to low vaccine usage in Paraná State. Multiple other studies have already observed those beneficial effects of vaccination at a population level, reinforcing the importance of a population-wide vaccination program to mitigate the effects of a newly emerging infectious disease [14,15,16].

Since distinct states and countries show distinct profiles of viral spread and disease lethality [17], which need to be even modeled in isolated state-wise scenarios [18], we tried to correlate Paraná state data to overall Brazilian one to access if the course of the local epidemic could be correlated to the nation-wide course (Figure 2c). We found a correlation between local weekly reported cases (Pearson = 0.8415), deaths (Pearson = 0.8778), and lethality (Pearson = 0.8042) to the overall country ones. This reinforces that our local scenario, despite being related with a single state and that epidemy transcurred differently in each Brazilian state, is positive correlated with the overall Brazil epidemiological scenario.

**Figure 3:**
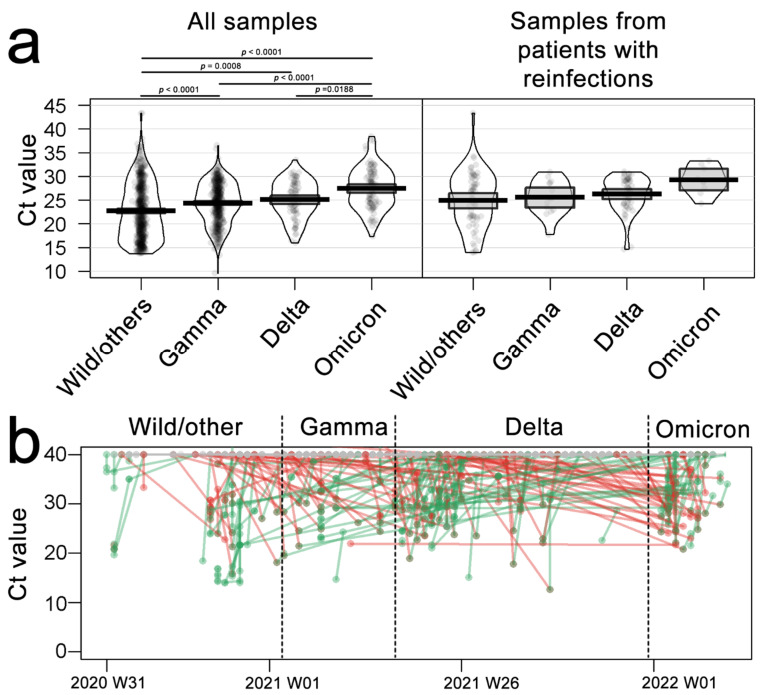
Cycle-threshold values for N gene target SARS-CoV 2 detection. (**a**) Ct values of diagnosis for each evaluated variant during the pandemic in all samples (left) or patients with multiple collections and reinfections. (**b**) Dots and lines plot represent Ct values for each patient tracked for multiple infections, gray lines represent negative results from the same patient, red lines represent Ct decrease (an increase in viral load), and green lines represent Ct increase (decrease in viral load). *p*-values derived from ANOVA, followed by Tukey’s test. Each dot represents a sample, the box represents the interquartile range, and the lines are the kernel-smoothed distribution.

We also evaluated the Ct values, representing the viral load, for the main observed variants (Figure 3). The Gamma variant viral load was lower than the Wild (*p* < 0.0001), Delta was lower than Gamma (*p* < 0.0001), and at last, Omicron was lower than Delta (*p* = 0.0188) (Figure 3a, left panel). This scenario resembled when restricted to patients with multiple collections (Figure 3a, right panel); however, there was no statistical support. Keeping the case-by-case strategy (Figure 3b), some reinfections showed an increase in the viral load. However, the overall scenario points to a reduction in the viral load during the diagnostic RT-PCR test, particularly for the Omicron variant, which is being observed by other authors as well [12,19].

Most cases attributed to the Omicron variant appear to be mild [20], with symptoms expected to be milder in vaccinated and those previously infected than in unvaccinated individuals [14]. This scenario has not increased the disease’s lethality, as observed in the previous Gamma outbreaks in Paraná, Brazil [2], enabling the reopening of schools, universities, and general crowd-related situations. However, even a milder infection can lead to an uncontrolled number of infections, which, in turn, will cause the overload of healthcare systems.

Based on numerous efforts of molecular tracing and sequence analysis, SARS-CoV-2 is shown to evolve in such a way as to determine subsequent waves of infection [17]. Rapid screening for target PCR–based diagnostic assays is essential for immediate public health strategies since new variants may still emerge. It was through target qPCR that the fast takeover of Delta and Omicron variants was detected, after predominance of Gamma in Curitiba-PR, southern Brazil. In conclusion, our data reinforce the rapid spread of VoCs in this region, highlighting the value and importance of agile and robust genomic surveillance systems, and sharing information with public health partners as such information is vital for the implementation of prevention strategies—namely, the use of masks, vaccination programs, COVID-19 testing, isolation, and others—that help prevent high mortality rates and the collapse of the healthcare system. 

## Data Availability

Not applicable.

## Supplementary Materials

The following supporting information can be downloaded at: https://www.mdpi.com/article/10.3390/v14040809/s1, Table S1: Data for Figure 1.
